# Application of low molecular weight heparins in umbilical artery thrombosis: A case series and review of the literature

**DOI:** 10.1097/MD.0000000000033501

**Published:** 2023-04-14

**Authors:** Ting Wang, Yingsha Yao, Ting Xu, Wenshan Wang, Yimin Zhou, Jing He, Ruoan Jiang

**Affiliations:** a Department of Obstetrics and Gynecology, Women’s Hospital Zhejiang University School of Medicine, Hangzhou, China; b Department of Pathology, Women’s Hospital Zhejiang University School of Medicine, Hangzhou, China; c Department of Ultrasonography, Women’s Hospital Zhejiang University School of Medicine, Hangzhou, China.

**Keywords:** case report, low molecular weight heparin, perinatal outcomes, umbilical artery thrombosis

## Abstract

Low molecular weight heparins are widely used in various thrombotic diseases and exert a preventive effect on thrombosis in high-risk patients. Umbilical artery thrombosis (UAT) is a rare occurrence that is difficult to detect during routine prenatal visits but can lead to adverse perinatal outcomes.

**Patient concerns:**

The aim of this study was to elucidate the therapeutic effect of low molecular weight heparins on UAT and to provide a new treatment option for the timing of delivery timing.

**Diagnoses and interventions:**

A retrospective study was conducted on cases involving thrombosis of the umbilical cord enrolled from July 2017 to July 2022. Data were acquired and analyzed from medical records and the final diagnosis was confirmed by histopathology. All included patients received LWMHs therapy after initial diagnosis of UAT.

**Outcomes:**

The mean age of the 10 pregnant women recruited into this study was 27.9 ± 4.0 year-of-age; 1 (10%) was elderly. The gestational age at diagnosis was 29.9 ± 3.7 weeks, the gestational age at termination was 36.3 ± 2.5 weeks and the mean gestational age of extension was 6.4 ± 4.2 weeks. Low molecular weight heparin sodium was administered after umbilical artery embolism was detected on ultrasound. The LWMHs treatment received by the included patients in this study was subcutaneous injection. The specific usage varies due to the types of LWMHs. Of the 10 cases, 5 (50%) had fetal distress but all fetuses were born alive without neonatal asphyxia. With regards to delivery mode, 9 pregnancies were terminated by cesarean section.

**Lesson:**

Early anticoagulant treatment with LWMHs may improve pregnancy outcomes. The timing and mode of termination of pregnancy should be determined according to the condition of the mother and the fetus along with the gestational age.

## 1. Introduction

Heparins and low molecular weight heparin (LMWHs) are the first choice for preventing and managing venous and arterial thromboembolic disease. Since early trials in the 1980s, a large amount of data has been published on the use of LMWHs to prevent and treat blood clots. The average molecular weight of unfractionated heparin (UFH) is much greater than that of the small heparin binding site of antithrombin or Factor Xa (58,000 Da), which is the target of anticoagulant effects. UFH-derived LMWHs are widely used because of their optimized molecular size, good efficacy and few side effects.^[1]^ For many indications, LWMHs are generally superior to UFH due to its good predictability, fewer side effects, and lower risk of induced bleeding.^[2]^ As a derivative of heparin, LMWHs are replacing heparin for traditional indications consequently.^[3,4]^

Umbilical artery thrombosis (UAT) is a rare occurrence. Thrombosis of one of the umbilical arteries may be associated with adverse pregnancy outcomes.^[5]^ Third trimester stillbirth arising from umbilical cord factors can occur occasionally and represents a significant challenge for obstetricians. At present, most literature relating to prenatal umbilical cord embolism are case reports; there are no case summaries relating to the use of and there is no case summary of LMWH for the treatment of UAT. In this study, we retrospectively analyzed 10 cases of singleton UAT who were treated with LWMHs in our hospital from July 2017 to July 2022. The characteristics of the disease were analyzed and the perinatal outcomes after LWMH intervention were summarized. Our intention was to provide reference guidelines for improving the understanding, diagnosis and treatment of this disease for obstetricians and gynecologists.

## 2. Materials and methods

Our hospital is an important rescue center for pregnant women, accepting all kinds of high-risk pregnant women referred by other hospitals. This study was approved by the Hospital Research Ethics Committee (Ethics reference: IRB-20220204-R).

### 2.1. Data extraction

Thirty-one cases with a diagnosis of UAT were retrieved from a database of 99,651 patients who gave birth between July 2017 and July 2022 at the Women Hospital School of Medicine at Zhejiang University. Ten of these cases received LWMHs after the ultrasound diagnosis of UAT.

### 2.2. Main standard of diagnosis

UAT is defined as a pregnant woman who has only 1 umbilical artery during the second and third trimesters and has a narrowed internal diameter of the other umbilical artery or no blood flow signal detected by ultrasound, but who have normal ultrasound and Doppler evaluations in the first or earlier second trimester.

Small for gestational age (SGA) is defined as: Understanding the relationship between birth weight, body length, head circumference, and gestational age; Below the 10th percentile.^[6]^

The umbilical coiling index (UCI) is defined as the number of umbilical coils divided by the length of the cord in centimeters. Normal UCI ranges from 0.07 to 0.30 twists/cm. Hypercoiling of the umbilical cord is usually defined as UCI > 0.30 twists/cm.^[7]^

A non-reactive NST is, by definition, an fetal heart rate monitoring interval that does not meet specific criteria.^[8–10]^ The criteria used to define reactive NST were at least 2 fetal heart rate accelerations, lasting at least 15 seconds, and at least 15 increases per minute from the established baseline heart rate. Most full-term fetuses have a lot of this acceleration during every 20 to 30 minutes of active sleep. Full-term fetuses rarely experience these accelerations for more than 60 minutes, and certainly never for more than 100 minutes that do not meet these criteria.

Decreased fetal movement count was defined as 10 fetal movement count within 2 hours.^[11]^

### 2.3. Pathology

Hematoxylin-Eosin stained umbilical cord sections of all patients in this study showed unilateral umbilical artery thrombosis. Hematoxylin-Eosin slides from all 10 cases were reviewed by 2 experienced placental pathologists.

## 3. Results

**Figure 2.**
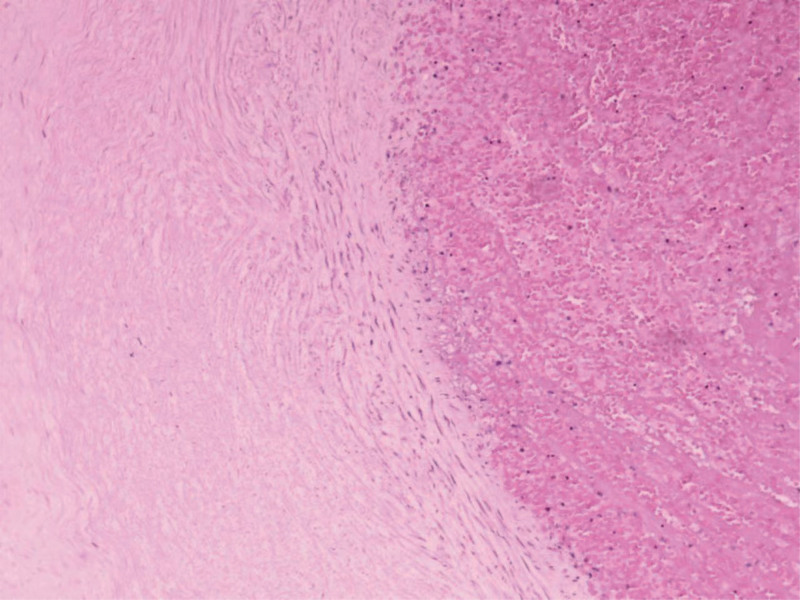
Umbilical arterial thrombosis demonstrates partial necrosis of the vascular wall and it was not accompanied by villous sclerosis or villous karyorrhexis, accompanied by inflammatory cell infiltration. The arterial lumen is filed with thrombi. Hematoxylin and Eosin, ×10 magnifcation (case 6).

Ten cases of UAT were identified; we retrieved the records for all 10 cases. All the 10 patients had ultrasonic and umbilical artery Doppler examination results. Typical Doppler findings are shown in Figure 1 (Case 10). Pathological examination after delivery confirmed the diagnosis of UAT; typical pathological manifestations are shown in Figure 2 (Case 6). LWMHs usage and maternal clinical data are shown in Table 1 and newborn characteristics are shown in Table 2.

**Table 1:** LWMHs usage and maternal characteristics.

No	Maternal age	Gravidity/parity	The time of diagnosis (weeks + days)	The days of pregnancy prolongation (days)	Underlying disease	pregnancy complication	D-dimer test before treatment (mg/L)	LWMHs varieties (Usage)			Aspirin	The last NST	Abnormal fetal movement(s)	Mode of delivery
								Varieties	Dosage	Frequency * days				
Case1	28	4/2	28 + 1	64	-	-	0.79	Fondaparinux sodium	2.5 mg	q.d*7	No	+	Increase	CS-selective
								Enoxaparin sodium	4000 iu	q.d*35				
Case2	26	1/0	31 + 3	40	-	-	1.59	Enoxaparin sodium	4000 iu	q.d*21 b.i.d*18	Yes	+	-	CS-selective
Case3	25	1/0	26 + 1	59	Hypothyroidism	-	10.33	Enoxaparin sodium	4000 iu	q.d*14	Yes	-	Decrease	CS-emergency
Case4	25	2/0	31	55	-	-	-	Enoxaparin sodium	4000 iu	q.d*42	Yes	+	-	VD
Case5	32	2/0	23	91	-	-	-	Nadroparin calcium	4100 iu	q.d*77 qod*13	No	-	-	CS-emergency
Case6	28	1/0	31 + 3	3	HBsAg+	-	1.6	Enoxaparin sodium	4000 iu	q.d*3	No	-	Decrease	CS-emergency
Case7	36	2/0	29 + 6	56	Myoma of uterus	Transient hypertension during pregnancy gestational diabetes mellitus	0.69	Nadroparin calcium	4100 iu	q.d*21	No	+	-	CS-selective
Case8	24	1/0	30 + 5	9	-	-	-	Enoxaparin sodium	4000 iu	q.d*9	Yes	-	Decrease	CS-emergency
Case9	31	2/0	31 + 6	40	-	-	1.22	Enoxaparin sodium	4000 iu	q.d*29	Yes	+	-	CS-selective
Case10	24	2/1	28 + 2	71	Congenital heart disease Ebstein malformation (tricuspid valve descent malformation)	PROM	1.12	Nadroparin calcium	4100 iu	q.d*24 q.o.d*44	No	+	-	CS-emergency

q.d: quaque die (every day).

b.i.d: bis in die (twice a day).

q.o.d: quaque omni die (every other day).

CS = cesarean section, VD =vaginal delivery, NST = prenatal non-stress test, PROM = premature rupture of membranes.

**Table 2:** Newborn characteristics (perinatal outcomes).

No	Gestational age (weeks + days)	Birth weight (g)	Gender	Apgar Score System (1/5 min)	Abnormality of umbilical cord	Abnormality of placenta	Characteristics of the amniotic fluid	Complications of newborn	Alive	Outcome
Case1	37 + 2	2300	Female	10–10	Cord around neck and body Hypercoiling	-	Clear	SGA	Yes	Rooming-in
Case2	37 + 1	2300	Female	10–10	Hypercoiling	Low-lying placenta, battledore cord insertion	Clear	SGA	Yes	Rooming-in
Case3	34 + 4	2170	Male	8–10	-	-	III	Gastroschisis (confirmed by prenatal examination)	Yes	Transfer to Children Hospital (operation for congenital deformity)
Case4	38 + 6	3000	Female	10–10	-	-	Clear	-	Yes	rooming-in
Case5	36	2750	Female	10–10	Hypercoiling	-	Clear	-	Yes	rooming-in
Case6	31 + 6	1600	Female	10–10	Hypercoiling (UCI = 0.4)	-	I	NRDS	Yes	39 days in hospital
Case7	37 + 6	2190	Male	10-10	Cord around neck Hypercoiling	Battledore cord insertion	Clear	SGA; Neonatal Hypoglycemia; NTP	Yes	6 days in hospital
Case8	32	1390	Female	9–10	Hypercoiling(UCI = 0.5)	-	Clear	SGA; neonatal wet lung; apnea of prematurity	Yes	49 days in our hospital, Transfer to Children Hospital (do not feed orally)
Case9	37 + 4	2100	Female	10–10	Stricture and Hypercoiling	-	I	SGA; Upper gastrointestinal malformation (confirmed by prenatal examination)	Yes	Transfer to Children Hospital (operation for congenital deformity)
Case10	38 + 3	2650	Male	10–10	-	-	II	SGA; HMD; Upper gastrointestinal malformation (confirmed by prenatal examination)	Yes	Transfer to Children Hospital (operation for congenital deformity)

HMD = hyaline membrane disease of newborn, NRDS = neonatal respiratory distress syndrome, NTP = Neonatal thrombocytopenia, SGA = small for gestational age, UCI = umbilical coiling index.

**Figrue 1.**
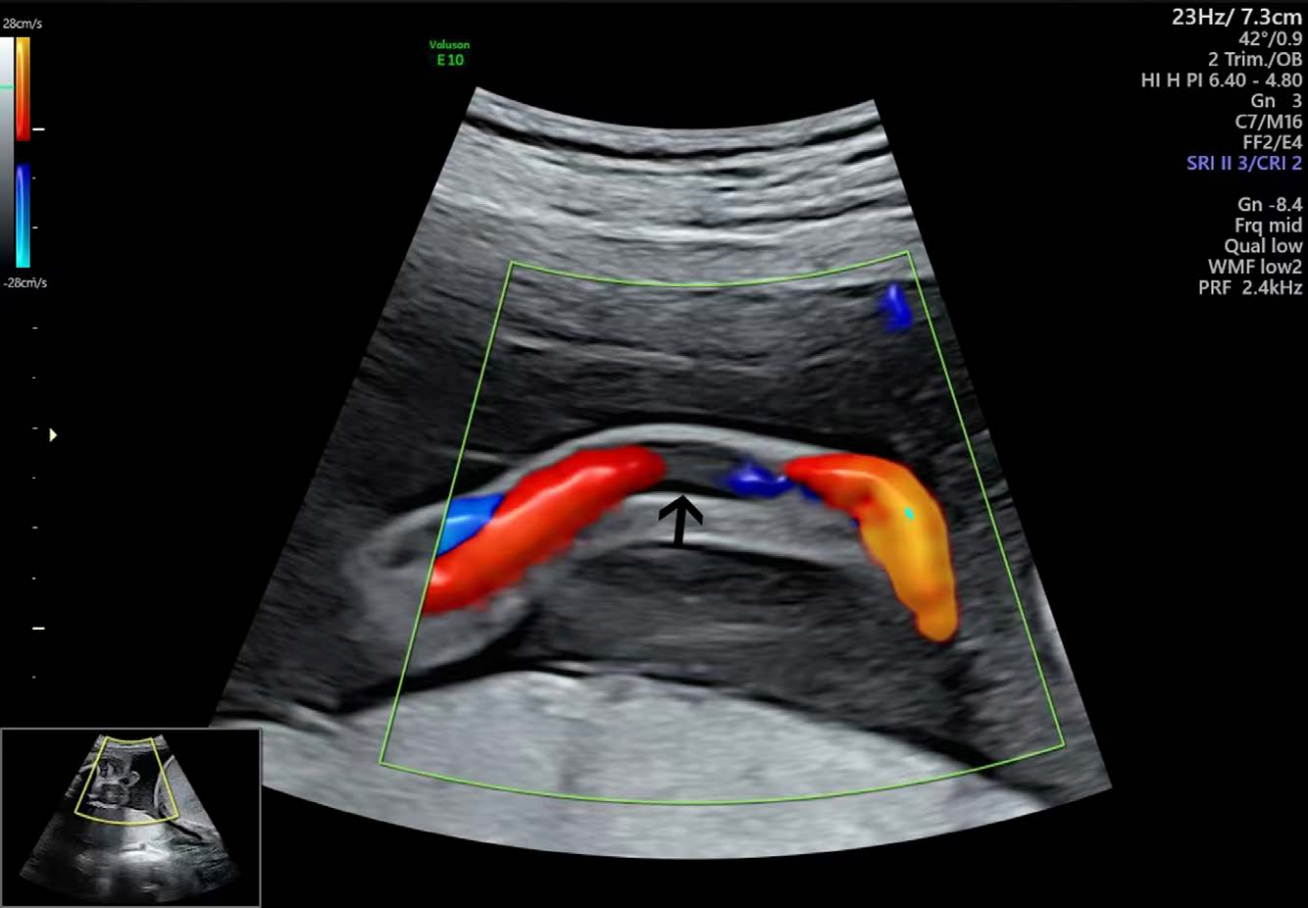
Single umbilical artery blood flow imaged using Doppler ultrasound at 28 + weeks (case 10), the scene of interruption of blood flow can be seen (arrow).

### 3.1. LWMHs usage and maternal characteristics

Half of the patients received LWMHs along with aspirin; the specific usage of LWMHs is shown in Table 1. The LWMHs treatment received by the included patients in this study was subcutaneous injection. The specific usage varies due to the types of LWMHs, as shown in Table 1. The mean maternal age was 27.9 ± 4.0 years. There were 8 primiparas (except for Case 1 and Case 10) and 4 primiparas (Cases 2, 3, 6, and 8). The mean gestational age of UAT was diagnosed as 29.9 ± 3.7 weeks and the median time of pregnancy prolongation was 55.5 (32.3–65.8) days. Four patients (Cases 3, 6, 7, and 10) had underlying diseases, including Case 7 with pregnancy complications. The levels of D-Dimers before treatment in Case 3 were significantly higher than the normal range. All cases except Case 4 were delivered by cesarean section.

### 3.2. Newborn characteristics (perinatal outcomes)

All 10 neonates were live births. The mean gestational age of the neonates was 36.3 ± 2.5 weeks and the mean birth weight was 2396.4 ± 685.0 g. The ratio of males to females was 3:7. All newborns had Apgar scores > 7 (no neonatal asphyxia). SGA was present in 60% of the newborns (Case 1, 2, 7, 8, 9, and 10). Three neonates with different degrees of fetal malformation (prenatal diagnosis) were referred to the Children Hospital for surgical correction after delivery. The other neonates were rooming-in or treated by the neonatal intensive care unit (NICU) according to the situation at birth.

Most umbilical cords and placentas were abnormal to varying degrees. Umbilical cord hypercoiling was present in 7 cases. The placentae of Case 2 and Case 7 were diagnosed as battledore placentae.

## 4. Discussion

Heparins and LMWHs are the first choice options for preventing and managing venous and arterial thromboembolic disease. Although low doses of heparin significantly reduce the incidence of perioperative deep vein thrombosis and fatal pulmonary embolism in surgical patients, the use of heparin is associated with increased hemorrhagic complications, especially hematoma, in perioperative patients receiving heparin.^[12]^ In order to develop safer and more convenient heparin preparations, a great deal of research has led to the development of low molecular weight heparin. These are heparin salts with molecular weights <8000 Da, and at least 60% of all chains have molecular weights <8000 Da.^[13]^ LMWHs contain specific 5-sugar sequences necessary for heparin to bind to antithrombin III, thereby promoting its subsequent enhancement and inhibition of activation Factor X. However, LMWHs are not long enough to bind and inhibit thrombin.^[14,15]^ Due to their biological and pharmacological properties, as well as their extensive evaluation and validation over the past 30 years, LMWHs have been well established for the prevention of thrombosis in high-risk surgical and medical patients and for the initial management of patients with venous thromboembolism.^[16]^ Since early trials in the 1980s, a great deal of data has been published on preventing and treating blood clots. In recent years, low molecular weight heparin has begun to replace heparin for traditional indications.^[3,4]^

Women are at an increased risk of both venous and arterial thromboembolism during pregnancy. Compared with women who were not pregnant,^[17]^ the risk of arterial thromboembolism (stroke and heart attack) increased by 3 to 4 times^[18,19]^ and a 4 to 5-fold increase in the risk of venous thromboembolism.^[20]^ Long-term use of LMWHs during pregnancy proved to be well tolerated, although the incidence of adverse effects was low.^[21–23]^ Santoro data appear to support the role of LMWHs in preventing further miscarriage in women with thrombotic disease and adverse history, and suggest that low molecular weight heparin may have therapeutic benefits that may temporarily improve maternal placental circulation parameters.^[24]^

UAT is a rare occurrence with an incidence estimated to be 1 case in every 16,600 gestations (0.006%)^[25]^ and up to 1 in every 250 deliveries (0.4%)^[26]^ when only high-risk pregnancies are taken into consideration. The incidence of UAT in our medical center is estimated to be 1 case in every 3200 single gestations (0.03%). UAT can lead to serious adverse consequences including fetal distress,^[25,27]^ stillbirth, fetal growth restriction, fetal organ infarction, neonatal cerebral palsy, and maternal-fetal blood transfusion. A soon as umbilical artery embolism is diagnosed, the termination of pregnancy may be the best option to avoid intrauterine fetal death; however, this may be associated with iatrogenic preterm birth. It is important to identify strategies to strengthen the monitoring of intrauterine fetal conditions and growth trends during pregnancy in order to prolong gestational age and reduce iatrogenic preterm birth while avoiding intrauterine stillbirth. In previous case reports, the timely termination of pregnancy or expectation was the main cause; drug intervention was rarely mentioned.

In our study, we reviewed the treatment process and outcome of UAT in treatment with LWMHs. All the newborns were born alive, including 4 cases of fetal distress and 6 cases of SGA. For Case 6, the newborn was admitted to the NICU for 39 days due to respiratory failure. The newborn of Case 7 was hospitalized in the neonatal intensive care unit for 6 days due to neonatal hypoglycemia; we considered that the neonatal hypoglycemia was related to the mother gestational diabetes mellitus. The neonate in Case 6 was admitted to the NICU for 39 days due to respiratory failure. The neonate in Case 8 spent 49 days in our NICU for neonatal wet lungs and apnea of prematurity and were then transferred to the Children Hospital due to problems associated with oral-feeding. The neonates in Cases 3, 9, and 10 were transferred to the Children Hospital to undergo surgeries to correct congenital deformities; all of these cases were congenital malformations found before birth.

The gestational ages of the series described in this review were small (some of them were mid-pregnancies). If a pregnancy was terminated immediately, the survival was usually very low. Prolonging the gestational age as much as possible is very important in order to improve the survival outcome of perinatal infants. Under the condition of close maternal and fetal observation, the timely termination of pregnancy can prolong the gestational age and increase the chances of survival for the fetus. All 10 cases in this study received treatment with LWMHs following the diagnosis of UAT by ultrasound; the median length of pregnancy prolongation was 55.5 (32.3–65.8) days. The mean prolongation of gestational age for the 11 UAT patients in our hospital during the same period was 4 (1–8) days; this was significantly shorter than for UAT patients treated with LWMHs (*P* = .002). We believe that the cases reviewed herein demonstrate that it is worth considering LWMH treatment for patients with UAT due to clear effects on the prolongation of gestational age. However, due to the small sample size, these findings need to be further confirmed in clinical practice. According to the current data, we believe that all types of LMWH can achieve certain effects. The appropriate selection of LWMH types should be considered comprehensively according to the patient condition and economic conditions. As for the cases included in this study, LWMHs treatment was safe for UAT patients, and no side effects of medication such as postpartum hemorrhage, hematoma, and neonatal coagulation disorder occurred in all cases. Although LWMHs treatment has a significant effect on prolonging gestational age and reducing stillbirth, neonatal SGA is still a common complication.

## 5. Conclusion

LWMHs is widely used in the prevention and treatment of thrombosis in various sites. The incidence of UAT is low but can lead to a high rate of neonatal adverse pregnancy outcomes. Once UAT is diagnosed, it is very effective to apply LWMH as soon as possible. In the case of UAT during pregnancy, the timing and mode of delivery should take into account the fetal morbidity associated with preterm birth, the possibility of fetal intolerance to delivery (fetal distress), and the risk of sudden adverse events.

## Author contributions

**Data curation:** Ting Wang, Yingsha Yao, Ting Xu, Wenshan Wang, Yimin Zhou.

**Formal analysis:** Ting Wang, Yingsha Yao, Wenshan Wang, Yimin Zhou.

**Supervision:** Jing He, Ruo-an Jiang.

**Writing – original draft:** Ting Wang, Yingsha Yao, Ting Xu, Yimin Zhou, Jing He, Ruo-an Jiang.

**Writing – review & editing:** Jing He, Ruo-an Jiang.
